# *N*-[(2*Z*,4*Z*)-4-Benzyl­idene-6-chloro-1,4-dihydro­pyrido[2,3-*d*][1,3]thia­zin-2-yl­idene]benzamide

**DOI:** 10.1107/S1600536812029741

**Published:** 2012-07-07

**Authors:** Manuel A. Fernandes, Demetrius C. Levendis, David H. Reid

**Affiliations:** a Molecular Sciences Institute, School of Chemistry, University of the Witwatersrand, Johannesburg, PO Wits 2050, South Africa

## Abstract

In the crystal structure of the title compound, C_21_H_14_ClN_3_OS, mol­ecules assemble into inversion dimers *via* pairs of N—H⋯N hydrogen bonds involving the N—H hydrogen of the thia­zine ring and the N atom of the pyridine ring. There is a close intra­molecular contact [2.570 (2) Å] between the carbonyl O atom of the benzamide and the S atom of the puckered thia­zine ring. The title compound can exist in two tautomeric forms, *viz.* amino or imino. The observed structure is compatible with the imino form on the basis of observed residual electron density and the two C—N bond lengths of 1.308 (2) and 1.353 (2) Å.

## Related literature

For the synthesis of related heterocycles, see: Fernandes & Reid (2003 ▶); Schmittel *et al.* (2004 ▶); Sonogashira *et al.* (1975 ▶). For related thia­zine structures, see: Cohen-Addad *et al.* (1981 ▶); Bernalte-Garcia *et al.* (2004 ▶); Kalman *et al.* (1987 ▶); Peng & Wu (2009 ▶); Palsuledesai *et al.* (2009 ▶).
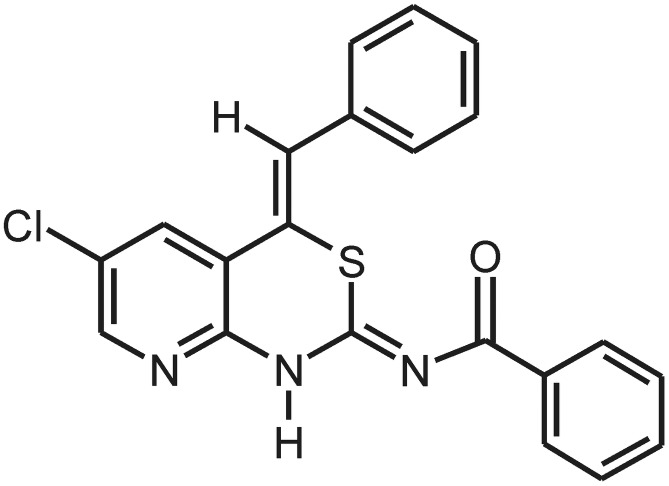


## Experimental

### 

#### Crystal data

C_21_H_14_ClN_3_OS
*M* *_r_* = 391.86
Triclinic, 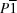

*a* = 7.2372 (2) Å
*b* = 8.3977 (3) Å
*c* = 15.7467 (6) Å
α = 101.227 (2)°
β = 98.427 (2)°
γ = 103.768 (1)°
*V* = 892.85 (5) Å^3^
*Z* = 2
Mo *K*α radiation
μ = 0.35 mm^−1^
*T* = 173 K
0.37 × 0.28 × 0.19 mm

#### Data collection

Bruker SMART APEXII CCD area-detector diffractometer
18107 measured reflections
3894 independent reflections
3538 reflections with *I* > 2σ(*I*)
*R*_int_ = 0.029

#### Refinement

*R*[*F* ^2^ > 2σ(*F* ^2^)] = 0.028
*wR*(*F* ^2^) = 0.077
*S* = 1.05
3894 reflections
248 parameters
7 restraints
H atoms treated by a mixture of independent and constrained refinement
Δρ_max_ = 0.27 e Å^−3^
Δρ_min_ = −0.21 e Å^−3^

### 

Data collection: *APEX2* (Bruker, 2005 ▶); cell refinement: *SAINT-Plus* (Bruker, 2005 ▶); data reduction: *SAINT-Plus* and *XPREP* (Bruker, 2005 ▶); program(s) used to solve structure: *SHELXS97* (Sheldrick, 2008 ▶); program(s) used to refine structure: *SHELXL97* (Sheldrick, 2008 ▶); molecular graphics: *PLATON* (Spek, 2009 ▶), *Mercury* (Macrae *et al.*, 2008 ▶) and *SCHAKAL99* (Keller, 1999 ▶); software used to prepare material for publication: *WinGX* (Farrugia, 1999 ▶) and *PLATON* (Spek, 2009 ▶).

## Supplementary Material

Crystal structure: contains datablock(s) global, I. DOI: 10.1107/S1600536812029741/nk2159sup1.cif

Structure factors: contains datablock(s) I. DOI: 10.1107/S1600536812029741/nk2159Isup2.hkl

Supplementary material file. DOI: 10.1107/S1600536812029741/nk2159Isup3.cml

Additional supplementary materials:  crystallographic information; 3D view; checkCIF report

## Figures and Tables

**Table 1:** Hydrogen-bond geometry (Å, °)

*D*—H⋯*A*	*D*—H	H⋯*A*	*D*⋯*A*	*D*—H⋯*A*
N2—H2⋯N3^i^	0.847 (16)	2.131 (17)	2.9733 (14)	173.1 (15)

Symmetry code: (i) 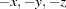.

**Fig. 1.**
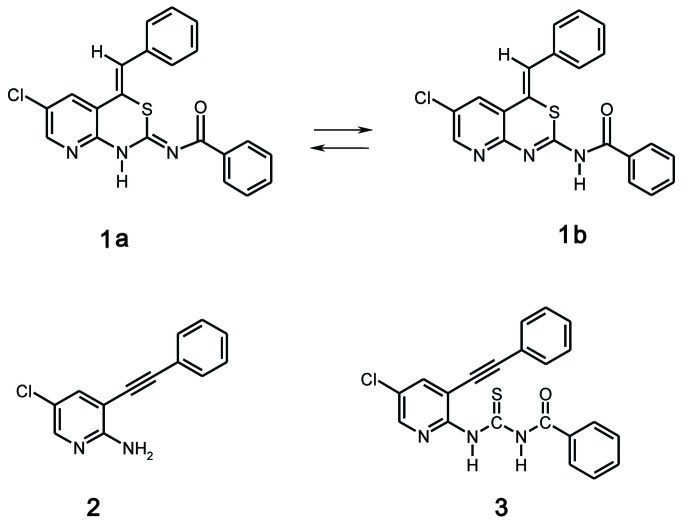
Two tautomeric forms of the title compound, the imino (1a) and amino (1 b) forms and reaction intermediates 2 and 3.

**Fig. 2.**
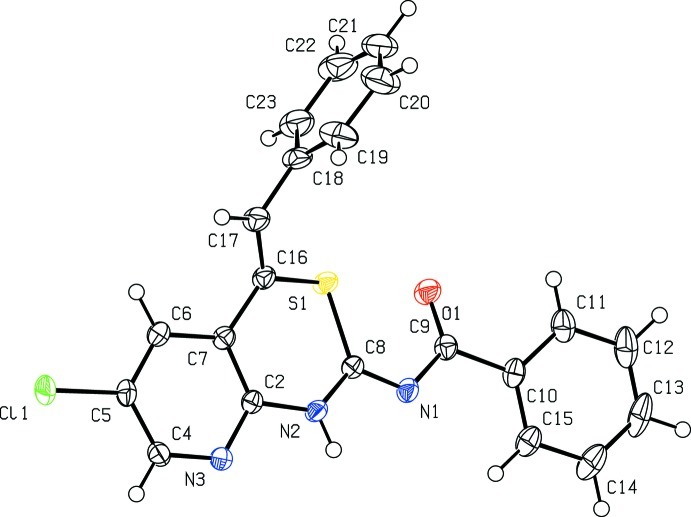
The molecular structure of the title compound, 1. Displacement ellipsoids are shown at the 50% probability level.

**Fig. 3.**
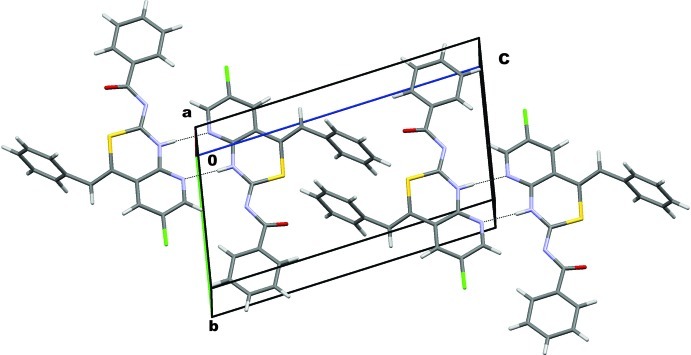
A view down the a axis of the unit cell showing the hydrogen-bonded dimers of the title molecule.

**Fig. 4.**
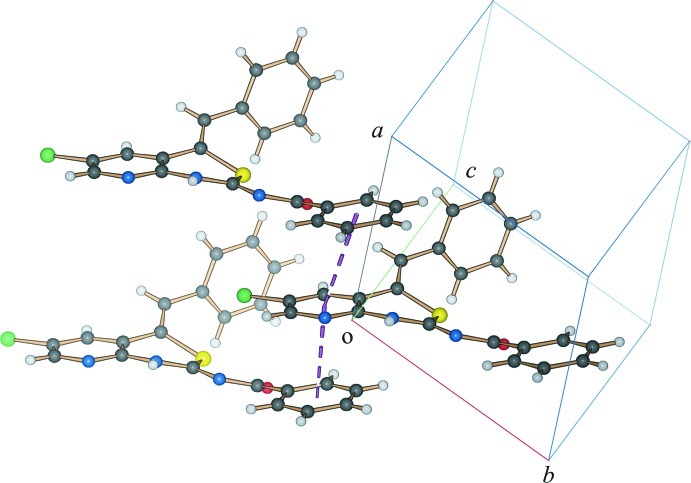
π–π interactions between the central pyridine ring and the benzoyl rings stacked along the a axis.

## supplementary crystallographic information

### Comment

The potential biological activity of 1,2-, 1,4-, 2,1- and 3,1 benzothiazines has stimulated the development of new syntheses of compounds based on these systems. Thus we (Fernandes & Reid, 2003) and other workers (Schmittel *et al*., 2004) have invented new syntheses of (4*Z*)-4-methylene-4*H*-3,1-benzothiazines. In furtherance of our studies we have applied the principle of our synthesis to the preparation of pyridine analogues of 4-methylene-4*H*-3,1-benzothiazines, namely 4-methylene-1,4-dihydropyrido[2,3-*d*] thiazines. Thus 2-amino-5-chloro-3-phenylethynylpyridine (2, Fig. 1), obtained by the Sonogashira reaction (Sonogashira *et al.*, 1975) of 2-amino-5-chloro-3-iodopyridine with phenylacetylene, reacted with benzoylisothiocyanate to give the thiourea (3, Fig. 1), which was cyclized with DBU to give the title compound. The structure of 1a has not been reported previously and is reported here (Fig. 2) as a part of an ongoing study of this class of 1,3-thiazines.

The title compound can possibly exist in two tautomeric forms, 1a and 1 b (Fig. 1). In the structure investigated in this work, residual electron density was observed about 0.85 Å from N(2), with no significant residual electron density near N(1), indicating that we have crystallized the imino form (1a). The C—N bond distances are also compatible with the imino form in which the C(8)—N(1) and C(8)—N(2) distances are 1.308 (2) and 1.353 (2) Å respectively. This is in agreement with a neutron diffraction study of the related 2-(2-chlorobenzoylimino)-1,3-thiazolidine (Cohen-Addad *et al.*, 1981). The intramolecular contact of 2.57 (2) Å between the carbonyl oxygen O(1) and S(1) is typical for imino thiazolidines (see for example Palsuledesai *et al.*, 2009). The molecules assemble *via* weak N—H···N hydrogen bonds into inversion dimers using the N—H hydrogen atom of the thiazine ring and the N of the pyridine group, with an N···N distance of 2.973 (1) Å. The packing of the hydrogen bonded dimers is shown in Fig. 3. Significant π–π interactions occur between the pyridine ring (N(3), C(2)—C(4)) at (*x*,*y*,*z*)) and the benzoyl ring (C10)-(15)) at either (*x* - 1, *y* - 1, *z*) or (*x*, *y* - 1, *z*)) with C*g*···C*g* distances of 3.651 (1) and 3.646 (1) Å respectively. This results in a stack of alternating pyridine and benzoyl rings interacting through π–π interactions along the *a* axis (Fig. 4).

### Experimental

The title compound (1a) was obtained by the reaction sequence:

2-amino-5-chloro-3-iodopyridine → (2) → (3) → (1).

For the preparation of 2-amino-5-chloro-3-phenylethynylpyridine, (2), phenylacetylene (6.0 ml, 54.6 mmol) was added to a stirred mixture of 2-amino-5-chloro-3-iodopyridine (12.72 g, 50 mmol), CuI (190 mg, 1 mmol) and (Ph_3_P)_2_PdCl_2_ (702 mg, 1 mmol) in Et_3_N (300 ml) under N_2_. After 24 h, the mixture was diluted with ether (300 ml) and filtered. Ether and Et_3_N were removed from the filtrate and the residual solid was dissolved in ether. The resulting solution was washed with water, dried, and solvent was removed. The residual solid was purified by chromatography on silica (CH_2_Cl_2_) to give (2) (9.72 g, 85%) as pale yellow crystals (m.p. 383-388 K).

For the preparation of *N*-nenzoyl-*N*'-(5-chloro-3-phenylethynylpyridyl-2)thiourea, (3), benzoylisothioisocyanate (0.74 ml, 5.5 mmol) was added to a solution of (2) (1.14 g, 5 mmol) in CH_2_Cl_2_ (10 ml) and the solution was kept at ambient temperature for 24 h. Hexane was added to complete the crystallisation that had partially taken place. The resulting solid was collected and purified by chromatography on silica (CH_2_Cl_2_), giving (3) (1.56 g, 79.6%) as lemon yellow crystals (m.p. 430-433 K).

For the preparation of *N*-[(2*Z*,4*Z*)-4-benzylidene-6-chloro-1,4-dihydropyrido[2,3-*d*][1,3]thiazin-2-ylidene]benzamide, (1), DBU (0.75 ml, 5 mmol) was added to a solution of (3) (784 mg, 2 mmol) in THF (10 ml). After 24 h the solid was chromatographed on silica (CH_2_Cl_2_ then 2% (V/V) EtOH in CH_2_Cl_2_). Solvent was removed from the eluates and the residual solid was recrystallized (DMF/MeCN) to give (1a) (631 mg, 80.5%; m.p. 502-504 K).

### Refinement

One N-bound H atom on the thiazine ring was placed according to the observed electron density and allowed to refine freely. The remaining H atoms were positioned geometrically and allowed to ride on their respective parent atoms, with C—H bond lengths of 1.00 (methine) and 0.99 Å (methylene CH_2_) and with *U*_iso_(H) = 1.2 times *U*_eq_(C). Phenyl atoms C21 and C22 were reported by *PLATON* to have slightly distorted atomic displacement (ADP) parameters. As a consequence, DELU and SIMU were used in the final refinements on the two atoms to restrain their ADPs to more reasonable values.

### Crystal data

**Table:**

C_21_H_14_ClN_3_OS	*Z* = 2
*M**_r_* = 391.86	*F*(000) = 404
Triclinic, *P*1	*D*_x_ = 1.458 Mg m^−^^3^
Hall symbol: -P 1	Mo *K*α radiation, λ = 0.71073 Å
*a* = 7.2372 (2) Å	Cell parameters from 6485 reflections
*b* = 8.3977 (3) Å	θ = 3.8–27°
*c* = 15.7467 (6) Å	µ = 0.35 mm^−^^1^
α = 101.227 (2)°	*T* = 173 K
β = 98.427 (2)°	Block, colourless
γ = 103.768 (1)°	0.37 × 0.28 × 0.19 mm
*V* = 892.85 (5) Å^3^	

### Data collection

**Table:**

Bruker SMART APEXII CCD area-detector diffractometer	*R*_int_ = 0.029
ω scans	θ_max_ = 27°, θ_min_ = 1.4°
18107 measured reflections	*h* = −8→9
3894 independent reflections	*k* = −10→10
3538 reflections with *I* > 2σ(*I*)	*l* = −20→20

### Refinement

**Table:**

Refinement on *F*^2^	7 restraints
Least-squares matrix: full	H atoms treated by a mixture of independent and constrained refinement
*R*[*F*^2^ > 2σ(*F*^2^)] = 0.028	*w* = 1/[σ^2^(*F*_o_^2^) + (0.0401*P*)^2^ + 0.2776*P*] where *P* = (*F*_o_^2^ + 2*F*_c_^2^)/3
*wR*(*F*^2^) = 0.077	(Δ/σ)_max_ = 0.001
*S* = 1.05	Δρ_max_ = 0.27 e Å^−^^3^
3894 reflections	Δρ_min_ = −0.21 e Å^−^^3^
248 parameters	

### Special details

**Table:**

Geometry. All e.s.d.'s (except the e.s.d. in the dihedral angle between two l.s. planes) are estimated using the full covariance matrix. The cell e.s.d.'s are taken into account individually in the estimation of e.s.d.'s in distances, angles and torsion angles; correlations between e.s.d.'s in cell parameters are only used when they are defined by crystal symmetry. An approximate (isotropic) treatment of cell e.s.d.'s is used for estimating e.s.d.'s involving l.s. planes.

### Fractional atomic coordinates and isotropic or equivalent isotropic displacement parameters (Å^2^)

**Table:**

	*x*	*y*	*z*	*U*_iso_*/*U*_eq_	
C2	−0.06525 (16)	−0.02208 (14)	0.13025 (7)	0.0204 (2)	
C4	−0.22472 (17)	−0.29516 (15)	0.05686 (8)	0.0240 (2)	
H4	−0.272	−0.3801	0.0033	0.029*	
C5	−0.25381 (17)	−0.33684 (15)	0.13550 (8)	0.0231 (2)	
C6	−0.18282 (17)	−0.21689 (15)	0.21447 (8)	0.0231 (2)	
H6	−0.2028	−0.2443	0.2688	0.028*	
C7	−0.08154 (16)	−0.05529 (14)	0.21279 (7)	0.0207 (2)	
C8	0.06508 (16)	0.28693 (14)	0.18128 (7)	0.0208 (2)	
C9	0.16648 (17)	0.57853 (15)	0.21226 (8)	0.0230 (2)	
C10	0.27176 (17)	0.72581 (15)	0.18204 (8)	0.0250 (3)	
C11	0.37511 (19)	0.87229 (16)	0.24628 (10)	0.0327 (3)	
H11	0.3827	0.8737	0.3072	0.039*	
C12	0.4664 (2)	1.01543 (18)	0.22140 (12)	0.0429 (4)	
H12	0.5375	1.1147	0.2651	0.051*	
C13	0.4540 (2)	1.01364 (18)	0.13303 (13)	0.0468 (4)	
H13	0.5156	1.1123	0.1161	0.056*	
C14	0.3525 (2)	0.86926 (19)	0.06890 (11)	0.0412 (4)	
H14	0.3446	0.8692	0.0081	0.049*	
C15	0.2619 (2)	0.72417 (17)	0.09306 (9)	0.0301 (3)	
H15	0.1937	0.6245	0.049	0.036*	
C16	0.01362 (18)	0.07578 (14)	0.29392 (8)	0.0229 (2)	
C17	0.0849 (2)	0.04176 (16)	0.36959 (8)	0.0285 (3)	
H17	0.0677	−0.0736	0.3706	0.034*	
C18	0.1887 (2)	0.17028 (16)	0.45202 (8)	0.0320 (3)	
C19	0.3837 (3)	0.1862 (2)	0.48353 (10)	0.0463 (4)	
H19	0.4457	0.1111	0.4536	0.056*	
C20	0.4890 (3)	0.3102 (2)	0.55806 (11)	0.0557 (5)	
H20	0.623	0.3217	0.5779	0.067*	
C21	0.3987 (3)	0.4163 (2)	0.60314 (10)	0.0548 (5)	
H21	0.4702	0.501	0.6543	0.066*	
C22	0.2053 (3)	0.3997 (2)	0.57427 (10)	0.0545 (5)	
H22	0.1429	0.4718	0.6063	0.065*	
C23	0.0990 (3)	0.27748 (19)	0.49810 (10)	0.0427 (4)	
H23	−0.0344	0.268	0.478	0.051*	
N1	0.12656 (14)	0.42335 (12)	0.15357 (6)	0.0230 (2)	
N2	0.02664 (15)	0.13768 (12)	0.12157 (7)	0.0225 (2)	
H2	0.052 (2)	0.144 (2)	0.0713 (11)	0.032 (4)*	
N3	−0.13207 (14)	−0.13887 (12)	0.05389 (6)	0.0224 (2)	
Cl1	−0.37769 (5)	−0.54060 (4)	0.13535 (2)	0.03250 (10)	
S1	0.03383 (5)	0.28939 (4)	0.290500 (18)	0.02461 (9)	
O1	0.11843 (14)	0.60154 (11)	0.28445 (6)	0.0292 (2)	

### Atomic displacement parameters (Å^2^)

**Table:**

	*U*^11^	*U*^22^	*U*^33^	*U*^12^	*U*^13^	*U*^23^
C2	0.0209 (5)	0.0187 (5)	0.0225 (5)	0.0056 (4)	0.0058 (4)	0.0062 (4)
C4	0.0236 (6)	0.0208 (6)	0.0242 (6)	0.0031 (4)	0.0016 (4)	0.0038 (5)
C5	0.0208 (5)	0.0179 (5)	0.0299 (6)	0.0026 (4)	0.0041 (4)	0.0082 (5)
C6	0.0252 (6)	0.0217 (6)	0.0250 (6)	0.0068 (5)	0.0068 (5)	0.0097 (5)
C7	0.0230 (5)	0.0189 (5)	0.0222 (5)	0.0072 (4)	0.0059 (4)	0.0067 (4)
C8	0.0217 (5)	0.0204 (5)	0.0214 (5)	0.0060 (4)	0.0054 (4)	0.0065 (4)
C9	0.0232 (6)	0.0203 (6)	0.0267 (6)	0.0066 (4)	0.0041 (4)	0.0083 (5)
C10	0.0237 (6)	0.0192 (6)	0.0358 (7)	0.0080 (5)	0.0104 (5)	0.0096 (5)
C11	0.0303 (7)	0.0219 (6)	0.0445 (8)	0.0052 (5)	0.0103 (6)	0.0056 (5)
C12	0.0358 (8)	0.0208 (6)	0.0703 (11)	0.0031 (6)	0.0198 (7)	0.0058 (7)
C13	0.0487 (9)	0.0244 (7)	0.0817 (12)	0.0115 (6)	0.0383 (9)	0.0255 (8)
C14	0.0520 (9)	0.0335 (8)	0.0541 (9)	0.0186 (7)	0.0295 (7)	0.0255 (7)
C15	0.0343 (7)	0.0246 (6)	0.0386 (7)	0.0114 (5)	0.0153 (6)	0.0142 (5)
C16	0.0294 (6)	0.0184 (5)	0.0225 (5)	0.0064 (5)	0.0085 (5)	0.0066 (4)
C17	0.0411 (7)	0.0215 (6)	0.0241 (6)	0.0085 (5)	0.0069 (5)	0.0078 (5)
C18	0.0530 (8)	0.0248 (6)	0.0194 (6)	0.0100 (6)	0.0064 (5)	0.0095 (5)
C19	0.0594 (10)	0.0467 (9)	0.0287 (7)	0.0188 (8)	−0.0033 (7)	0.0032 (6)
C20	0.0656 (11)	0.0570 (11)	0.0324 (8)	0.0097 (9)	−0.0085 (8)	0.0047 (8)
C21	0.0885 (14)	0.0394 (9)	0.0233 (7)	0.0007 (9)	0.0015 (8)	0.0049 (6)
C22	0.0980 (15)	0.0359 (8)	0.0330 (8)	0.0197 (9)	0.0264 (9)	0.0047 (7)
C23	0.0629 (10)	0.0373 (8)	0.0313 (7)	0.0159 (7)	0.0163 (7)	0.0086 (6)
N1	0.0261 (5)	0.0188 (5)	0.0246 (5)	0.0043 (4)	0.0069 (4)	0.0074 (4)
N2	0.0304 (5)	0.0184 (5)	0.0191 (5)	0.0043 (4)	0.0083 (4)	0.0062 (4)
N3	0.0246 (5)	0.0202 (5)	0.0214 (5)	0.0042 (4)	0.0041 (4)	0.0054 (4)
Cl1	0.03448 (18)	0.02067 (15)	0.03662 (18)	−0.00357 (12)	0.00425 (13)	0.00945 (13)
S1	0.03781 (18)	0.01751 (15)	0.02036 (15)	0.00759 (12)	0.00947 (12)	0.00618 (11)
O1	0.0408 (5)	0.0218 (4)	0.0268 (4)	0.0083 (4)	0.0106 (4)	0.0074 (4)

### Geometric parameters (Å, º)

**Table:**

C2—N3	1.3365 (15)	C12—H12	0.95
C2—N2	1.3908 (15)	C13—C14	1.383 (2)
C2—C7	1.3968 (16)	C13—H13	0.95
C4—N3	1.3374 (15)	C14—C15	1.3901 (18)
C4—C5	1.3822 (17)	C14—H14	0.95
C4—H4	0.95	C15—H15	0.95
C5—C6	1.3797 (17)	C16—C17	1.3379 (17)
C5—Cl1	1.7319 (12)	C16—S1	1.7768 (12)
C6—C7	1.3877 (16)	C17—C18	1.4821 (18)
C6—H6	0.95	C17—H17	0.95
C7—C16	1.4669 (16)	C18—C23	1.386 (2)
C8—N1	1.3075 (15)	C18—C19	1.391 (2)
C8—N2	1.3531 (15)	C19—C20	1.388 (2)
C8—S1	1.7641 (12)	C19—H19	0.95
C9—O1	1.2300 (15)	C20—C21	1.375 (3)
C9—N1	1.3818 (15)	C20—H20	0.95
C9—C10	1.4912 (16)	C21—C22	1.371 (3)
C10—C15	1.3896 (18)	C21—H21	0.95
C10—C11	1.3970 (18)	C22—C23	1.399 (2)
C11—C12	1.384 (2)	C22—H22	0.95
C11—H11	0.95	C23—H23	0.95
C12—C13	1.378 (3)	N2—H2	0.847 (16)
			
N3—C2—N2	114.40 (10)	C15—C14—H14	119.9
N3—C2—C7	123.77 (10)	C10—C15—C14	119.61 (13)
N2—C2—C7	121.83 (10)	C10—C15—H15	120.2
N3—C4—C5	122.00 (11)	C14—C15—H15	120.2
N3—C4—H4	119	C17—C16—C7	123.17 (11)
C5—C4—H4	119	C17—C16—S1	118.99 (9)
C6—C5—C4	120.21 (11)	C7—C16—S1	117.84 (8)
C6—C5—Cl1	119.72 (9)	C16—C17—C18	124.98 (11)
C4—C5—Cl1	120.07 (9)	C16—C17—H17	117.5
C5—C6—C7	118.52 (11)	C18—C17—H17	117.5
C5—C6—H6	120.7	C23—C18—C19	118.71 (14)
C7—C6—H6	120.7	C23—C18—C17	122.21 (14)
C6—C7—C2	117.58 (10)	C19—C18—C17	119.07 (13)
C6—C7—C16	122.17 (10)	C20—C19—C18	120.99 (16)
C2—C7—C16	120.20 (10)	C20—C19—H19	119.5
N1—C8—N2	116.67 (10)	C18—C19—H19	119.5
N1—C8—S1	123.60 (9)	C21—C20—C19	119.80 (18)
N2—C8—S1	119.73 (9)	C21—C20—H20	120.1
O1—C9—N1	124.89 (11)	C19—C20—H20	120.1
O1—C9—C10	119.63 (11)	C22—C21—C20	120.00 (15)
N1—C9—C10	115.47 (10)	C22—C21—H21	120
C15—C10—C11	119.70 (12)	C20—C21—H21	120
C15—C10—C9	122.27 (11)	C21—C22—C23	120.59 (16)
C11—C10—C9	117.95 (11)	C21—C22—H22	119.7
C12—C11—C10	120.15 (14)	C23—C22—H22	119.7
C12—C11—H11	119.9	C18—C23—C22	119.87 (17)
C10—C11—H11	119.9	C18—C23—H23	120.1
C13—C12—C11	119.86 (14)	C22—C23—H23	120.1
C13—C12—H12	120.1	C8—N1—C9	118.58 (10)
C11—C12—H12	120.1	C8—N2—C2	127.76 (10)
C12—C13—C14	120.48 (13)	C8—N2—H2	115.3 (11)
C12—C13—H13	119.8	C2—N2—H2	116.6 (11)
C14—C13—H13	119.8	C2—N3—C4	117.83 (10)
C13—C14—C15	120.19 (14)	C8—S1—C16	101.63 (5)
C13—C14—H14	119.9		
			
N3—C4—C5—C6	−1.49 (19)	S1—C16—C17—C18	−2.56 (19)
N3—C4—C5—Cl1	179.12 (9)	C16—C17—C18—C23	64.2 (2)
C4—C5—C6—C7	−0.37 (18)	C16—C17—C18—C19	−114.62 (16)
Cl1—C5—C6—C7	179.02 (9)	C23—C18—C19—C20	−2.0 (2)
C5—C6—C7—C2	2.81 (17)	C17—C18—C19—C20	176.88 (15)
C5—C6—C7—C16	−174.49 (11)	C18—C19—C20—C21	1.8 (3)
N3—C2—C7—C6	−3.79 (17)	C19—C20—C21—C22	−0.2 (3)
N2—C2—C7—C6	177.37 (11)	C20—C21—C22—C23	−1.2 (3)
N3—C2—C7—C16	173.57 (11)	C19—C18—C23—C22	0.6 (2)
N2—C2—C7—C16	−5.27 (17)	C17—C18—C23—C22	−178.26 (13)
O1—C9—C10—C15	−153.18 (12)	C21—C22—C23—C18	1.0 (2)
N1—C9—C10—C15	25.39 (17)	N2—C8—N1—C9	179.63 (10)
O1—C9—C10—C11	23.54 (17)	S1—C8—N1—C9	−1.12 (16)
N1—C9—C10—C11	−157.89 (11)	O1—C9—N1—C8	−13.03 (18)
C15—C10—C11—C12	0.3 (2)	C10—C9—N1—C8	168.49 (10)
C9—C10—C11—C12	−176.46 (12)	N1—C8—N2—C2	−170.01 (11)
C10—C11—C12—C13	0.5 (2)	S1—C8—N2—C2	10.70 (17)
C11—C12—C13—C14	−0.6 (2)	N3—C2—N2—C8	162.12 (11)
C12—C13—C14—C15	0.0 (2)	C7—C2—N2—C8	−18.93 (19)
C11—C10—C15—C14	−1.03 (19)	N2—C2—N3—C4	−179.06 (10)
C9—C10—C15—C14	175.64 (12)	C7—C2—N3—C4	2.02 (17)
C13—C14—C15—C10	0.9 (2)	C5—C4—N3—C2	0.68 (17)
C6—C7—C16—C17	29.87 (19)	N1—C8—S1—C16	−165.12 (10)
C2—C7—C16—C17	−147.36 (13)	N2—C8—S1—C16	14.12 (11)
C6—C7—C16—S1	−150.07 (10)	C17—C16—S1—C8	146.10 (11)
C2—C7—C16—S1	32.70 (15)	C7—C16—S1—C8	−33.96 (10)
C7—C16—C17—C18	177.51 (12)		

### Hydrogen-bond geometry (Å, º)

**Table:**

*D*—H···*A*	*D*—H	H···*A*	*D*···*A*	*D*—H···*A*
N2—H2···N3^i^	0.847 (16)	2.131 (17)	2.9733 (14)	173.1 (15)

Symmetry code: (i) −*x*, −*y*, −*z*.
